# Recurrent Corneal Erosion Induced by 1% 5-Fluorouracil Solution

**DOI:** 10.7759/cureus.73238

**Published:** 2024-11-07

**Authors:** Hayato Tanaka, Hitoshi Tabuchi

**Affiliations:** 1 Ophthalmology, Tsukazaki Hospital, Himeji, JPN; 2 Ophthalmology, Hiroshima University, Hiroshima, JPN

**Keywords:** 5-fluorouracil, chemo-holidays, conjunctival papilloma, corneal epithelial erosion, cytotoxic anticancer drugs

## Abstract

Cytotoxic anticancer drug solution eye drops can hinder the cell division of corneal basal cell division and corneal limbal epithelial stem cell migration. We discuss a case involving recurrent corneal epithelial erosions induced by 1% 5-fluorouracil (5-FU) solution eye drops. A 68-year-old male with conjunctival papilloma on his left eye underwent recurrences three times and additional tumor excision. He was prescribed 1% 5-FU solution eye drops two times a day without drug holidays and experienced recurrent corneal epithelial erosion. This report highlights the necessity of drug holidays for patients receiving cytotoxic anticancer drug solution eye drops.

## Introduction

5-fluorouracil (5-FU) is a pyrimidine analog, and it blocks DNA and RNA synthesis and can suppress the cell division of corneal epithelial basal cells [1]. 1% 5-FU solution eye drops are commonly used for treating ocular surface squamous neoplasia [2,3]. Chemo holidays - planned pauses in chemotherapy treatment - should be scheduled during treatment with 1% 5-FU solution eye drops to give ocular surface epithelial cells a chance to recover. Dosing cycles that involve a one-week dosing period and three-week chemo holidays are generally considered a good practice [2]. Common side effects of 5-FU solution eye drops include pain and redness of the ocular surface, superficial punctate keratopathy, filamentous keratitis, and eyelid swelling [4,5].

Keizer et al. have reported two cases of benign papillomas treated with 1% 5-FU solution eye drops four times daily without chemo holidays [6], which had to be discontinued due to local corneal and conjunctival epithelial erosions and ectropion of the eyelid. There is only one report involving the application of 1% 5-FU solution to treat conjunctive papilloma. The prognosis of conjunctive papilloma treated with 5-FU application has not been clearly elucidated. This report describes a case with repetitive corneal erosion induced by 1% 5-FU solution eye drops without chemo holidays. It aims to provide insights into the mechanism of drug-induced corneal epithelial damage and the importance of chemo holidays when applying cytotoxic chemotherapy eye drops for ocular surface tumors.

This article was previously presented as a poster at the 39th Asia-Pacific Academy of Ophthalmology (APAO) Congress on February 22-25, 2024.

## Case presentation

**Figure 1:**
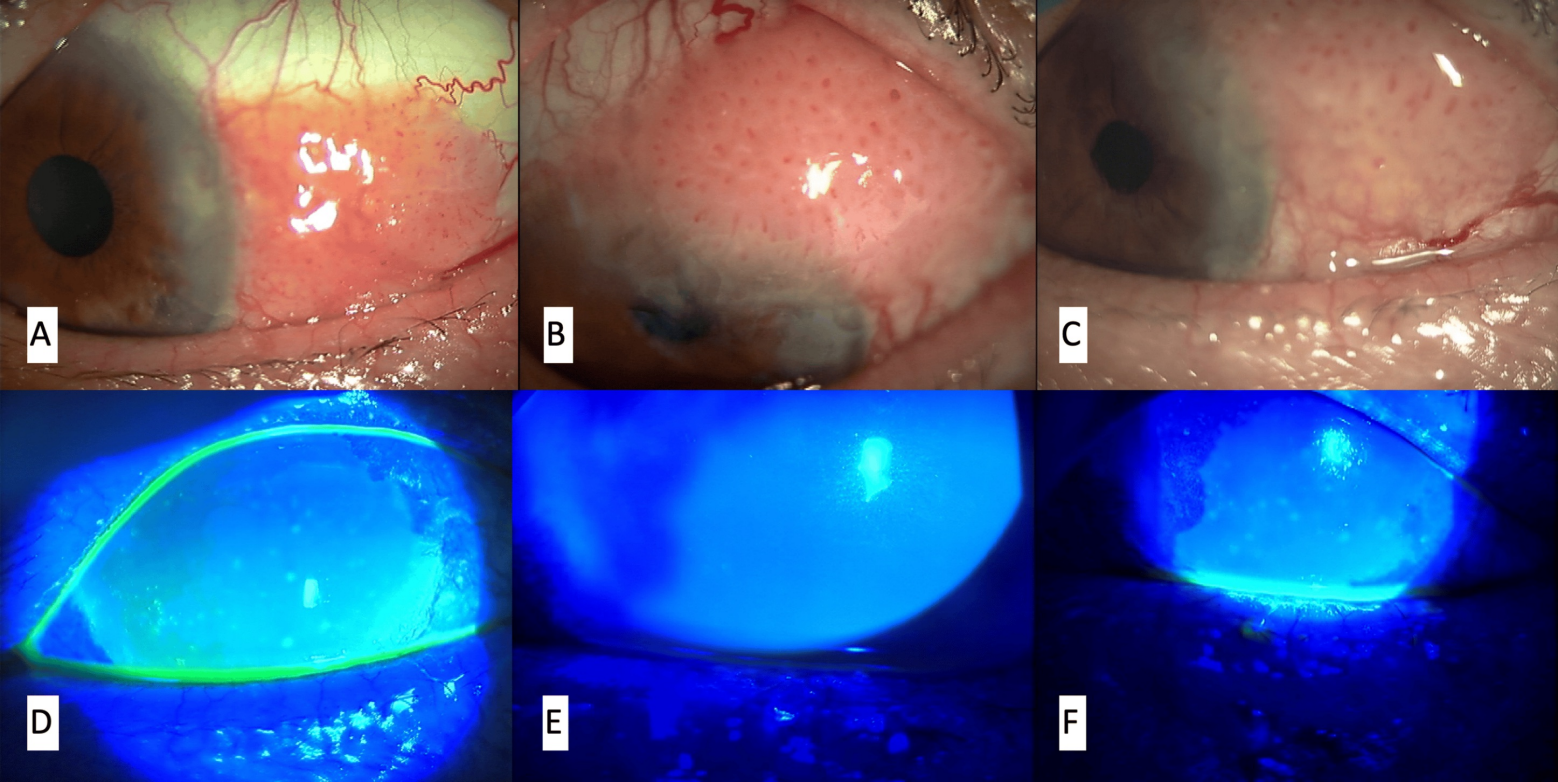
Slit-lamp microscopy photographs of the anterior segment A-C: Taken before each tumor excision: June 2018 (A), May 2021 (B), February 2023 (C). D-F: Taken when corneal epithelial erosion occurred during the administration of 1% 5-FU solution without chemo holidays: May 2019 (A), April 2020 (B), April 2023 (C)

A 68-year-old male with a conjunctival tumor in his left eye was referred to our hospital in March 2018. The first excision and pathological evaluation revealed that the conjunctival tumor was papilloma, but it repeatedly recurred. Additional excisions with mitomycin C were performed three times: in June 2018, May 2021, and February 2023. The patient started adjunctive chemotherapy with 1% 5-FU solution eye drops in April 2019. The regimen consisted only of morning and evening 1% 5-FU solution per day with no washout period. After continuously using the 1% 5-FU solution eye drops for one month, the left eye developed corneal epithelial erosion. The patient discontinued the 1% 5-FU solution eye drops and covered the erosion with soft contact lenses. The erosion resolved within a month. The patient resumed the 1% 5-FU solution eye drops as a treatment for tumor recurrence in March 2020 and March 2023. However, the erosion reappeared both times and resolved only after the eye drops were discontinued. After the last erosion epithelialization, the patient was followed up without 1% 5-FU solution, and the conjunctival papilloma had yet to recur until August 2024. Figure 1 shows slit-lamp microscopy images of the anterior segment.

## Discussion

This case report highlights the importance of drug holidays in the application of cytotoxic anticancer drugs, especially 5-FU. Thoft et al. have described the homeostasis of the corneal epithelium as a balance (X+Y=Z) between the sum (X+Y) of basal cell division (X) and limbal epithelial stem cell migration (Y), and the desquamation of squamous epithelial cells (Z) [7]. Corneal erosion is thought to occur when a state equivalent to X+Y<Z in the former explanation persists for an extended period in the corneal epithelium.

Cytotoxic anticancer drugs, including 5-FU, are typically administered with scheduled drug holidays. The proportion of cells in a mitotic rest state, which is also less susceptible to cytotoxic anticancer drugs, is higher in normal cell populations. Therefore, after administration, a difference in survival rates emerges between tumor cell populations and normal cell populations. Subsequently, by repeating the process of waiting for normal cell populations to recover during drug holidays before re-administration, the difference in survival rates can be gradually widened [8].

In the present case, the absence of chemo holidays continuously accelerated corneal epithelial squamous cell shedding and inhibited corneal epithelial basal cell division, leading to corneal erosions. The two cases discussed by Keizer et al. [6] also received 5-FU eye drops without drug holidays. Our findings align with those of Keizer et al. and highlight the importance of drug holidays to prevent adverse effects. To the best of our knowledge, there are no reports comparing continuous administration regimens of 5-FU eye drops and regimens with drug holidays in terms of adverse events in the literature. The superiority of regimens with drug holidays cannot be established at present with respect to adverse events. Hence, we recommend further studies to more comprehensively investigate whether 5-FU application with adequate chemo holiday is effective in conjunctive papilloma as adjunctive therapy when the papilloma is resistant to tumor excision.

## Conclusions

We presented a case where corneal erosion developed during the twice-daily application of 1% 5-FU solution eye drops, which epithelialized after the discontinuation of the drops. Application of cytotoxic anticancer eye drops without drug holidays can suppress not only tumor cells but also normal epithelial basal cells. This report emphasizes the importance of scheduling chemo holidays when prescribing cytotoxic chemotherapy eye drops, including 1% 5-FU solution.
